# Parameter optimization of IPT systems based on genetic algorithms

**DOI:** 10.1371/journal.pone.0360165

**Published:** 2026-10-08

**Authors:** Jin Guo, Langtao Xing

**Affiliations:** 1 School of Economics and Management, Shangluo University, Shangluo, Shaanxi, China; 2 Aviation Engineering School, Air Force Engineering University, Xi’an, Shaanxi, China; University of Botswana, BOTSWANA

## Abstract

Output power and transmission efficiency are key performance indicators for Wireless Power Transfer (WPT). Inductive Power Transfer (IPT) systems feature numerous resonant parameters that are interrelated, making it challenging to determine specific component values during system design. This paper investigates an S-P type IPT system and establishes an equivalent circuit model for the mutual inductance of the contactless transformers. The influence characteristics of coupling coefficient *k*, resonant angular frequency *ω*, and equivalent load resistance *R*_e_ on circuit output power and transmission efficiency are analyzed. By setting the efficiency-power product as the objective function and *k*, *ω*, and *R*_e_ as decision variables, this paper innovatively transforms the parameter design problem of the IPT system into a nonlinear parameter optimization problem. This approach not only enables the fitness function to better escape local optima. By adjusting the duty cycle of the Buck-Boost circuit, the equivalent load resistance is optimized to its optimal value. Optimize the parameters *k*, *ω*, and *R*_e_ to maximize the system’s efficiency-power product output. Through parameter design, the IPT system attains a globally optimal design for both output power and transmission efficiency, resolving the challenge of determining parameters for resonant compensation structures.

## 1. Introduction

Wireless Power Transfer (WPT) technology is an integrated approach that leverages power electronics and modern control theory to achieve wireless electrical energy transmission via electric or magnetic fields. This technology has become a research hotspot both domestically and internationally. Inductive Power Transfer (IPT) systems are widely applied in the field of wireless charging for electric vehicles, demonstrating broad application prospects [1–3].

Output power and transmission efficiency are critical performance parameters for evaluating IPT systems. Parameters such as the non-contact transformer specifications, resonant frequency, coupling coefficient, equivalent output load resistance, and resonant network parameters all influence output power and transmission efficiency [4]. Fluctuations in the positional offset between the primary and secondary coils of the non-contact transformer are common, primarily manifesting as vertical displacement, horizontal displacement, and spatial angular displacement, as illustrated in Fig 1. The structure of the non-contact transformer determines parameters such as the coupling coefficient, self-inductance, and mutual inductance. Therefore, the optimized design of the non-contact transformer is another critical factor affecting the transmission efficiency and output power of wireless charging. These factors can all cause variations in the coupling coefficient. The equivalent load resistance changes during the charging process for different load objects. The accuracy of the resonant capacitor components also affects the output characteristics. Significant variations in IPT system circuit parameters can cause substantial fluctuations in output voltage, current, power, and transmission efficiency characteristics, complicating control and potentially leading to system instability in severe cases [5,6]. Consequently, optimizing the circuit parameters of IPT systems remains a current research challenge.

**Fig 1:**
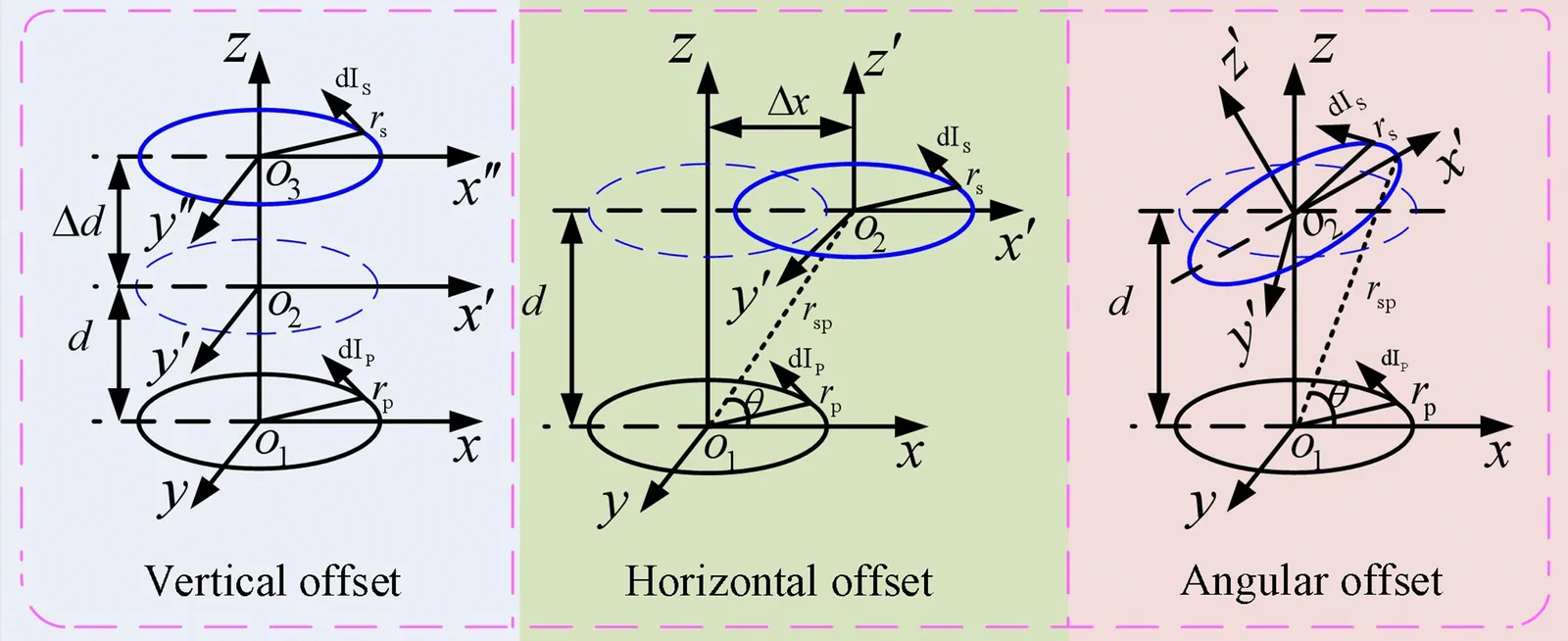
Coil space offset.

To address the challenge of designing circuit parameters for IPT systems, researchers have conducted in-depth investigations and studies from various perspectives. In [7], the non-contact transformer coil was optimized to maintain a constant coupling coefficient during axial or lateral displacement. In [8], the output characteristics of the non-contact transformer’s lateral offset capability were enhanced by employing an optimized compensation capacitor method. In [9–12], an equivalent magnetic circuit model for contactless transformers was constructed to optimize their structural design and enhance wireless power transfer performance. In [13], the impact of variations in sensitivity parameters—such as load, mutual inductance, and capacitance deviation—on wireless charging systems was thoroughly analyzed. The study also demonstrated that impedance matching circuits can maximize power transfer efficiency. In [14,15], under coil spatial displacement conditions, the effects of load and quality factor on the output characteristics of a wireless charging system were analyzed, and the system parameter design was optimized. The aforementioned methods analyze the impact of circuit parameters on wireless charging systems from various perspectives and employ diverse techniques to optimize the structure of contactless transformers and circuit parameter design. While these approaches have improved the performance of wireless charging systems to a certain extent, several issues remain worthy of further refinement.

Additionally, some scholars have combined multi-objective functions with nonlinear optimization algorithms to perform parameter optimization design within IPT systems. Genetic algorithms (GA) can handle complex combinatorial problems with faster convergence rates and higher efficiency. The application of genetic algorithms for optimizing WPT systems has emerged in recent years [16–18]. In [19], genetic algorithms were employed to optimize the coil, but the impact of optimization process details on the results was overlooked. In [20], a genetic algorithm was applied in two steps to design a resonant WPT system operating in the megahertz range. First, the algorithm extracted equivalent circuit parameters, then determined suitable values for the variable capacitor to eliminate frequency splitting. In [21], an optimization method based on a universal algorithm is proposed. During the design iteration, the coil track layout is interpreted using binary strings, and practical considerations and specific implementation methods for the optimization process are introduced. In [22], the receiving coil was optimized through a genetic algorithm, resulting in improved coupling performance. The proposed system achieved optimal transmission efficiency. In [23], a multi-objective particle swarm optimization algorithm is introduced for designing ring couplers to enhance wireless power transfer performance. The aforementioned scholars primarily conducted parameter analysis and optimization design targeting coupled structures, maximum output power, or efficiency enhancement, without comprehensively considering output power and transmission efficiency metrics for global system optimization. To address this gap, this paper employs the efficiency-power product as the objective function and *k*, *ω*, and *R*_e_ as decision variables to achieve global optimal parameter design for IPT systems.

In this work, based on the S-P type IPT system under study, an equivalent circuit model for non-contact transformer mutual inductance is established. First, the influence characteristics of coupling coefficient *k*, resonant angular frequency *ω*, and equivalent load resistance *R*_e_ on circuit output power and transmission efficiency are analyzed. Subsequently, by treating the efficiency product as the objective function and *k*, *ω*, and *R*_e_ as decision variables, the parameter design problem of the IPT system is innovatively transformed into a nonlinear parameter optimization problem. The equivalent load resistance is optimized by adjusting the duty cycle of the Buck-Boost circuit, achieving the maximum efficiency-power product output through parameter tuning of *k*, *ω*, and *R*_e_. Finally, an experimental wireless charging platform was constructed to validate the feasibility of the theoretical analysis. This work demonstrates the significant theoretical value of genetic algorithm-based parameter optimization design, while also providing a foundation for designing adaptive automatic tracking systems and enhancing the performance of wireless charging systems.

The remainder of this paper is organized as follows: Section II analyzes the output characteristics of power and transmission efficiency for S-P type IPT systems and presents the analytical process for the maximum efficiency-power product objective. Section III details the specific process of genetic algorithm-based parameter optimization for the IPT system. Section IV constructs a wireless power transmission device to validate the theoretical analysis. The concluding section summarizes the research findings.

## 2. Basic principles of the IPT system

Based on the inductive coupling method for power transmission, non-contact transformers in wireless charging circuits suffer from drawbacks such as high leakage inductance and low coupling coefficient. Therefore, resonant networks must be incorporated to enhance transmission efficiency and output power. Common resonant compensation structures include S-S, S-P, P-S, and P-P configurations, where the first S or P denotes series or parallel compensation on the primary side, and the second S or P indicates series or parallel compensation on the secondary side. The S-P compensation network offers the advantage of a significantly larger output resonant current. This paper primarily analyzes the S-P type resonant compensation circuit for wireless charging.

### 2.1. Analysis of system transmission efficiency and power

To simplify the analysis of the IPT system, Fig 2 shows the main circuit of the electric vehicle IPT system. In the figure, the DC voltage *U*_i_ of the inverter circuit on the *U*_i_ side is replaced by a constant voltage source. Switching transistors *S*_1_ ~ *S*_4_ form a high-frequency H-bridge inverter circuit. *L*_p_ and *L*_s_ represent the primary and secondary self-inductances of the transformer, respectively. *M* denotes the mutual inductance between the primary and secondary windings. Capacitors *C*_1_ and *C*_2_ constitute the resonant capacitors of the compensation topology. Diodes *D*_1_–*D*_4_ form the secondary-side uncontrolled rectifier. *L*_1_, *S*_5_, *D*_5,_ and *C*_4_ form the Buck-Boost converter, with *U*_bat_ representing the output battery voltage. Z_in_ denotes the input equivalent impedance. *R*_e_ is the equivalent load resistance of the secondary rectifier circuit, and *R*_L_ is the equivalent internal resistance of the battery.

**Fig 2:**
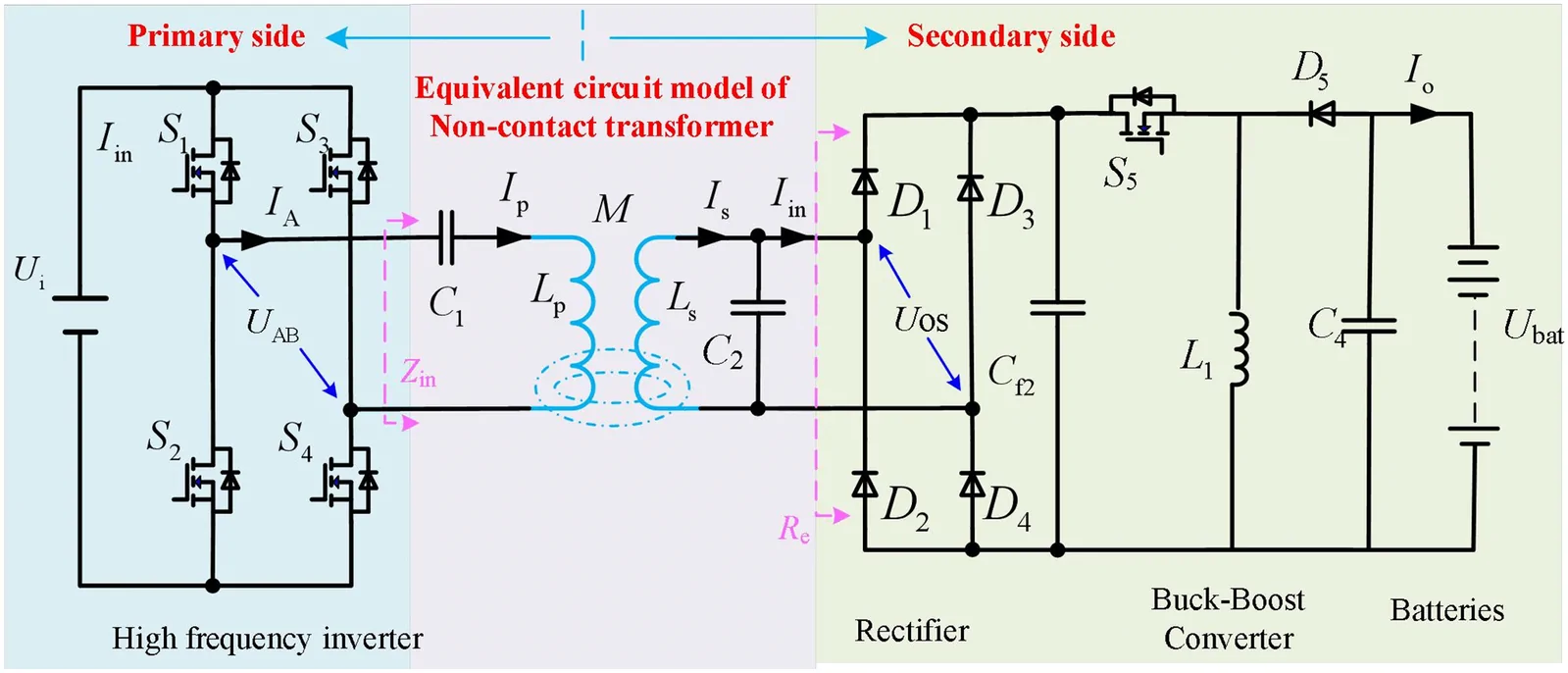
Main circuit of electric vehicle IPT system.

To simplify circuit analysis, the *M* model of the non-contact transformer in the S-P compensation network is shown in Fig 3. The resonant capacitance of the S-P resonant network satisfies $\omega_{\text{0}}=1/\sqrt{L_{\text{p}}C_{\text{1}}}=1/\sqrt{L_{\text{s}}C_{\text{2}}}$.

**Fig 3:**
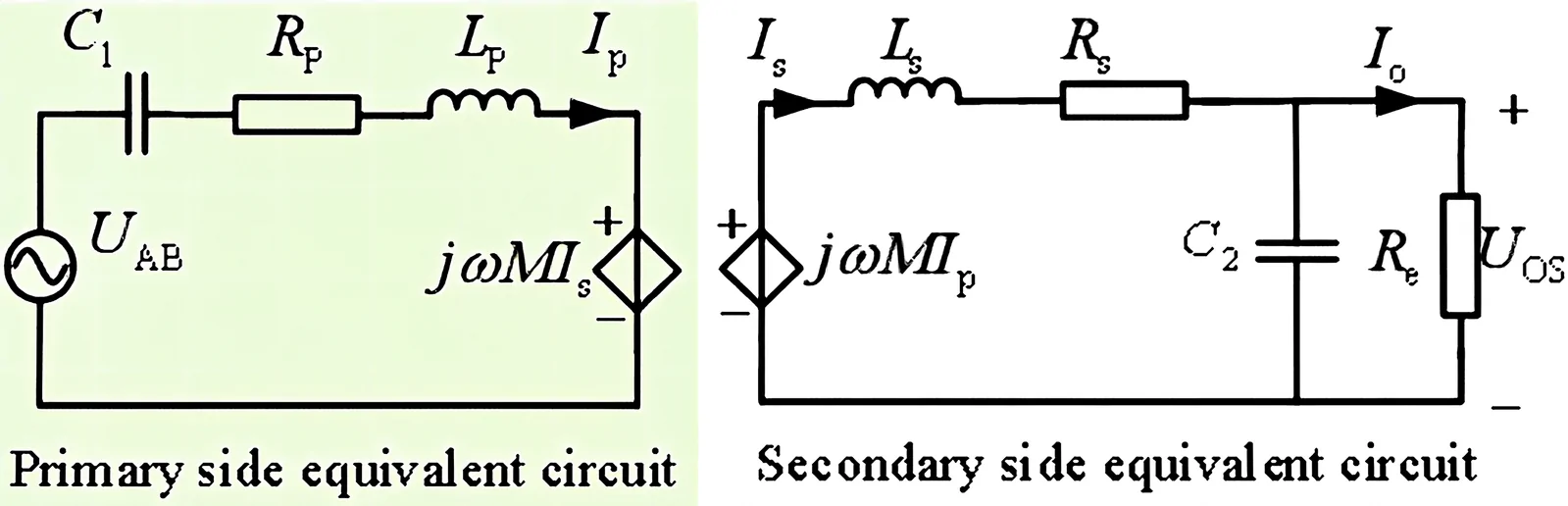
S-P compensation circuit mutual inductance model.

Based on the circuit mutual inductance model, the circuit equations for the S-P resonant compensation network derived from Kirchhoff’s laws are as follows:


$$\begin{array}{c} \left\{ \begin{array}{c} @c@{\mathrm{\backslash stackrel}•U}_{\text{AB}}={\mathrm{\backslash stackrel}•I}_{\text{p}}Z_{\text{1}}-jX_{\text{M}}{\mathrm{\backslash stackrel}•I}_{\text{s}}\\ 0={\mathrm{\backslash stackrel}•I}_{\text{s}}Z_{\text{2}}-jX_{\text{M}}{\mathrm{\backslash stackrel}•I}_{\text{p}} \end{array}\right. \end{array}$$ (1)

Where $Z_{\text{1}}=R_{\text{1}}+j\text{(}X_{\text{PL}}+X_{\text{PL}}\text{)}$, $Z_{\text{2}}=R_{\text{2}}+{jX}_{\text{SL}}+\frac{{jX}_{\text{SC}}R_{\text{e}}}{\left(R_{\text{e}}+{jX}_{\text{SC}}\right)}$, $X_{\text{PL}}={\omega L}_{\text{P}}$, $X_{\text{PC}}=-\frac{\text{1}}{{\omega C}_{\text{1}}}$, $X_{\text{M}}=\omega M$, $X_{\text{SL}}=\omega L_{\text{s}}$, $X_{\text{SC}}=-\frac{\text{1}}{{\omega C}_{\text{2}}}$, Z_1_ and Z_2_ are the equivalent impedances of the primary and secondary sides, respectively. *X*_PL_ and *X*_SL_ are the equivalent inductive reactances of the primary and secondary sides, respectively. *X*_PC_ and *X*_LC_ are the equivalent capacitive reactances of the primary and secondary sides, respectively. *k* is the coupling coefficient of the non-contact transformer, which can be expressed as $k=X_{\text{M}}/\sqrt{X_{\text{PL}}X_{\text{PS}}}$.

From Eq. (1), the primary and secondary currents of the circuit can be solved as follows:


$$\begin{array}{c} \left\{ \begin{array}{c} @c@{\mathrm{\backslash stackrel}•I}_{\text{p}}=\frac{{\mathrm{\backslash stackrel}•U}_{\text{AB}}Z_{\text{2}}}{Z_{\text{1}}Z_{\text{2}}+kX_{\text{PL}}^{\text{2}}X_{\text{PS}}^{\text{2}}}\\ {\mathrm{\backslash stackrel}•I}_{\text{s}}=\frac{jX_{\text{M}}{\mathrm{\backslash stackrel}•U}_{\text{AB}}}{Z_{\text{1}}Z_{\text{2}}+kX_{\text{PL}}^{\text{2}}X_{\text{PS}}^{\text{2}}} \end{array}\right. \end{array}$$ (2)

From Eq. (1), the equivalent load power received and transmission efficiency of the resonant compensation network can be solved as follows:


$$\begin{array}{c} P=\frac{{U_{\text{AB}}}^{\text{2}}X_{\text{PL}}^{\text{2}}X_{\text{PS}}^{\text{2}}X_{\text{SC}}^{\text{2}}kR_{\text{e}}}{\left(X_{\text{SC}}^{\text{2}}+{R_{\text{e}}}^{\text{2}})(A^{\text{2}}+B^{\text{2}}\right)} \end{array}$$ (3)

$$\begin{array}{c} \eta =\frac{kR_{\text{e}}X_{\text{PL}}^{\text{2}}X_{\text{PS}}^{\text{2}}}{(1+{R_{\text{e}}}^{\text{2}}/X_{\text{SC}}^{\text{2}})\left[A\left(R_{\text{s}}+E)+B(X_{\text{SL}}+\text{E}R_{\text{e}}/X_{\text{SC}}\right)\right]} \end{array}$$ (4)

Where $\text{A=[}R_{\text{p}}\text{(}R_{\text{s}}\text{+E)-(}X_{\text{PL}}\text{+}X_{\text{PC}}\text{)(}X_{\text{SL}}\text{+E}R_{\text{e}}/X_{\text{SC}}\text{)+}kX_{\text{PL}}^{\text{2}}X_{\text{PS}}^{\text{2}}\text{]}$, $\text{E=}X_{\text{SC}}^{\text{2}}R_{\text{e}}/(R_{\text{e}}+X_{\text{SC}}^{\text{2}})$,

$\text{B=[}R_{\text{p}}\text{(}X_{\text{SL}}\text{+E}R_{\text{e}}/X_{\text{SC}}\text{)+(}X_{\text{PL}}\text{+}X_{\text{PC}}\text{)(}R_{\text{s}}\text{+E)]}$.

From Eqs. (3) and (4), it can be seen that the output power and transmission efficiency of the IPT system are influenced by multiple parameters in the circuit, including frequency, coupling coefficient, resonant inductance, resonant capacitance, and output equivalent load. Based on Eqs. (3) and (4), the circuit’s performance curves as a function of coupling coefficient, angular frequency, and output equivalent resistance are shown in Fig 4. It can be observed that near the resonant angular frequency *ω*_₀_, the output power and efficiency of the resonant compensation network reach their maximum values. From Fig 4(a) and (c), it is evident that an optimal equivalent resistance *R*_e_ and an optimal coupling coefficient *k* exist to maximize the output power. In Fig 4(b), the transmission efficiency first increases and then decreases with changes in the equivalent resistance. Fig 4(d) shows that the transmission efficiency initially increases as the coupling coefficient increases.

**Fig 4:**
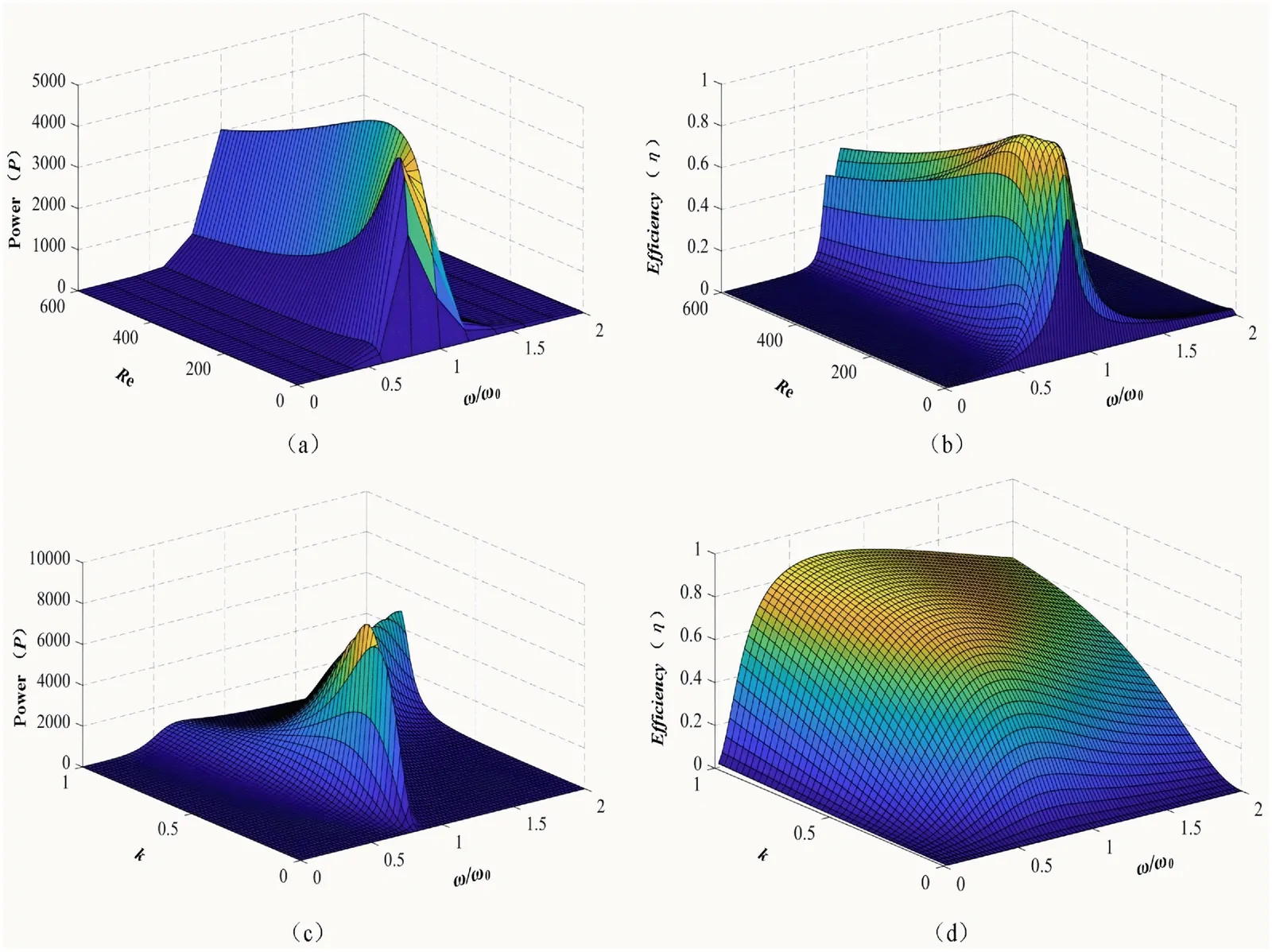
Relationship between coupling coefficient, angular frequency, output equivalent impedance parameters, and circuit performance: (a) Relationship between output power and angular frequency versus output equivalent impedance; (b) Relationship between transmission efficiency and angular frequency versus output equivalent impedance; (c) Relationship between output power and angular frequency versus coupling coefficient; (d) Relationship between transmission efficiency and angular frequency versus coupling coefficient.

### 2.2. System parameter optimization for maximum efficiency-power product transmission objective

The equivalent load resistance *R*_e_ and coupling coefficient *k* influence the output power and transmission efficiency of the resonant compensation network. Let *ω* denote the operating angular frequency of the inverter and *ω*_0_ denote the resonant angular frequency of the S-P resonant network. The inverter frequency variation range is defined as *ω*_min_ ≤ *ω* ≤ *ω*_max_. The S-P resonant network can operate in three resonant states. The three circuit resonance modes are: under-resonance mode (*ω* < *ω*_0_), fully resonant mode (*ω* = *ω*_0_), and over-resonance mode (*ω* > *ω*_0_). Within the inverter’s frequency conversion range, the circuit operates in three resonance modes: ① Under-resonance mode (*ω*_min_ ≤ *ω* < *ω*_0_), ② Full-resonance mode (*ω* = *ω*_0_), ③ Over-resonance mode (*ω*_0_ < *ω* ≤ *ω*_max_). Based on Eqs. (3) and (4), the curves showing how output power and transmission efficiency vary with the coupling coefficient *k* are depicted in Fig 5.

**Fig 5:**
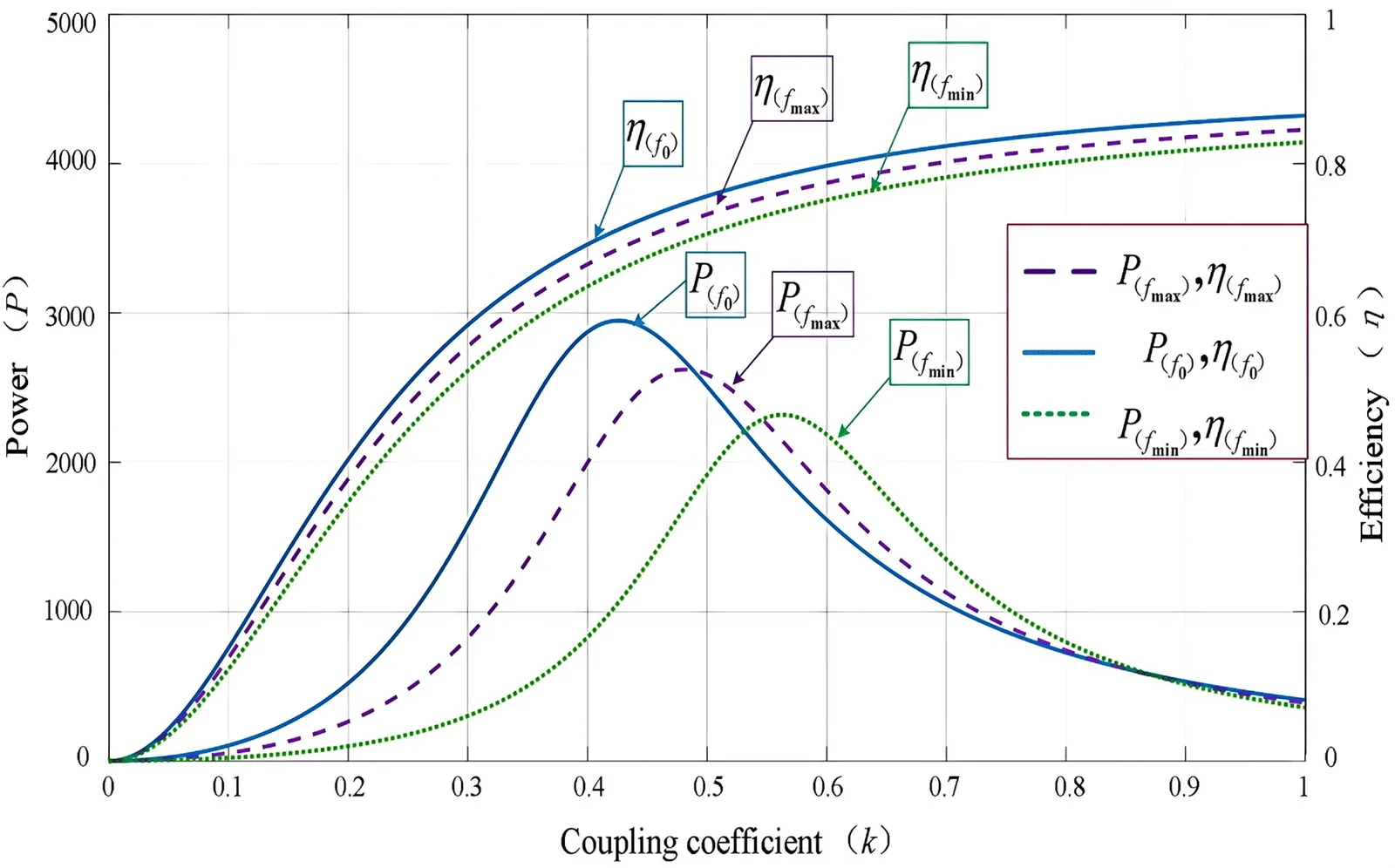
Output power and transmission efficiency vs. coupling coefficient.

The coupling coefficient parameter is influenced by factors such as the non-contact transformer coupling structure, transmission distance, and the offset positions of the primary and secondary coils [24]. Fig 5 shows that under operating conditions *ω*_min_, *ω*_0_, and *ω*_max_, the inverter output power exhibits an optimal coupling coefficient *k*_opt_ that maximizes the resonant network’s output power. Transmission efficiency increases with the coupling coefficient. According to Eq. (3), setting the derivative of *P*_(k)_ to zero yields the optimal coupling coefficient.


$$\begin{array}{c} \frac{dP\text{(}k\text{)}}{dk}=0 \end{array}$$ (5)

According to Eq. (5), the optimal coupling coefficient *k*_opt_ and maximum output power *P*_max_ under the circuit’s maximum output power condition can be described as:


$$\begin{array}{c} k_{\text{opt}}=\sqrt{\frac{R_{\text{p}}\left[R_{\text{s}}^{\text{2}}X_{\text{SC}}^{\text{2}}+{\left(X_{\text{SL}}X_{\text{SC}}+R_{\text{s}}R_{\text{e}}\right)}^{\text{2}}\right]}{X_{\text{PL}}X_{\text{SL}}\left[X_{\text{SC}}^{\text{2}}\left(R_{\text{s}}+R_{\text{e}}\right)+R_{\text{s}}^{\text{2}}R_{\text{e}}^{\text{2}}\right]}} \end{array}$$ (6)

$$\begin{array}{c} P_{\text{max}}=P\text{(}k_{\text{opt}}\text{)}=\frac{U_{\text{AB}}^{\text{2}}R_{\text{e}}}{4R_{\text{p}}\left[X_{\text{SC}}^{\text{2}}\left(R_{\text{s}}+R_{\text{e}}\right)+R_{\text{s}}^{\text{2}}R_{\text{e}}^{\text{2}}\right]} \end{array}$$ (7)

This paper considers both transmitted power and transmission efficiency, employing the efficiency-power product to optimize the coupling coefficient parameters of the S-P compensation topology circuit. The efficiency-power product is a comprehensive evaluation metric that integrates both system output power and transmission efficiency. The optimization approach for the efficiency-power product primarily focuses on system output power as the design objective [25,26]. Normalize the system output power. Define ζ as the system load factor. Set ζ as the ratio of the system’s actual output power to its maximum output power, i.e., $\zeta =P\text{(}k\text{)}/P_{\text{max}}$ the efficiency-power product *λ* can be defined as the product of the system load factor ζ and efficiency *η*, denoted as λ=ζη. The expression for the system load factor and efficiency-power product *λ* is:


$$\begin{array}{c} \zeta =\frac{P\text{(}k\text{)}}{P_{\text{max}}}=\frac{4R_{\text{p}}kX_{\text{PL}}X_{\text{SL}}\text{[}R_{\text{S}}^{\text{2}}\text{+(}X_{\text{SL}}\text{-}R_{\text{S}}R_{\text{e}}/X_{\text{SC}}{\text{)}}^{\text{2}}\text{][}R_{\text{S}}+R_{\text{e}}\text{+}R_{\text{S}}R_{\text{e}}^{\text{2}}/X_{\text{SC}}^{\text{2}}\text{]}}{[R_{\text{S}}R_{\text{P}}+R_{\text{P}}(X_{\text{SL}}+R_{\text{S}}R_{\text{e}}/X_{\text{SC}}{)}^{\text{2}}+k^{\text{2}}X_{\text{PL}}X_{\text{SL}}(R_{\text{S}}+R_{\text{e}}\text{+}R_{\text{S}}R_{\text{e}}^{\text{2}}/X_{\text{SC}}^{\text{2}}){]}^{\text{2}}} \end{array}$$ (8)

$$\begin{array}{c} \lambda =\zeta \eta =\frac{4X_{\text{PL}}X_{\text{SL}}R_{\text{e}}R_{\text{P}}(R_{\text{S}}+R_{\text{e}}\text{+}R_{\text{S}}R_{\text{e}}^{\text{2}}/X_{\text{SC}}^{\text{2}})\left[R_{\text{S}}^{\text{2}}\text{+(}X_{\text{SL}}\text{-}R_{\text{S}}R_{\text{e}}/X_{\text{SC}}{\text{)}}^{\text{2}}\right]}{\{k^{\text{2}}X_{\text{PL}}X_{\text{SL}}(R_{\text{S}}+R_{\text{e}}\text{+}R_{\text{S}}R_{\text{e}}^{\text{2}}/X_{\text{SC}}^{\text{2}})+[R_{\text{S}}^{\text{2}}\text{+(}X_{\text{SL}}\text{-}R_{\text{S}}R_{\text{e}}/X_{\text{SC}}{\text{)}}^{\text{2}}]{\}}^{\text{3}}} \end{array}$$ (9)

According to Eq. (9), the calculated curve of the efficiency-power product *λ* versus coupling coefficient *k* is shown in Fig 6. With the primary and secondary coil self-inductances, resonant capacitance, resonant angular frequency, and equivalent input resistance parameters fixed, it can be observed that the efficiency-power product first increases and then decreases as the coupling coefficient increases. An optimal coupling coefficient exists that maximizes the efficiency-power product. Fig 7 illustrates the variation of the efficiency-power product with angular frequency and output equivalent impedance. It is evident that the efficiency-power product reaches its maximum near the resonant angular frequency. As the load resistance increases, the efficiency-power product first increases and then decreases.

**Fig 6:**
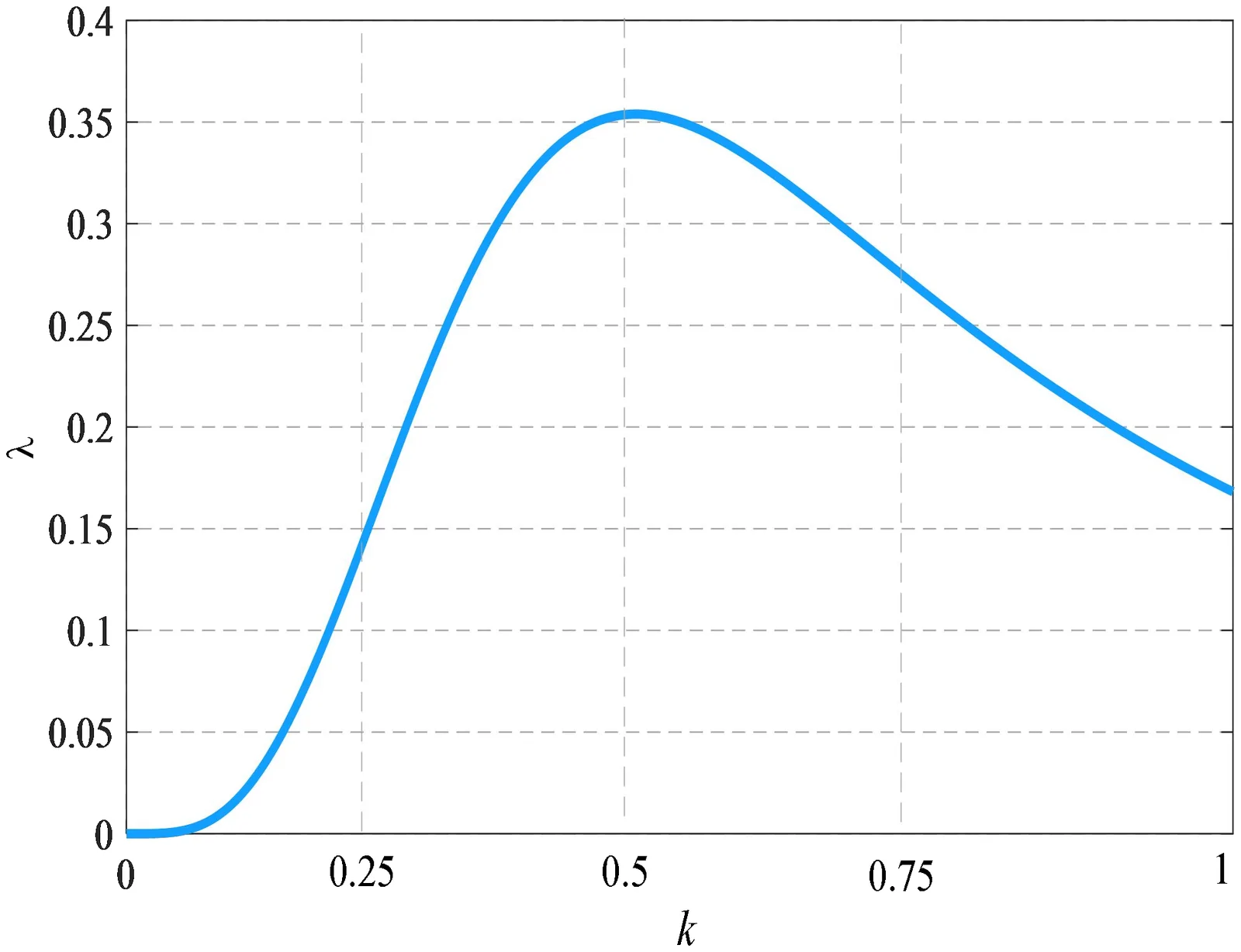
Efficiency-power product vs. coupling coefficient curve.

**Fig 7:**
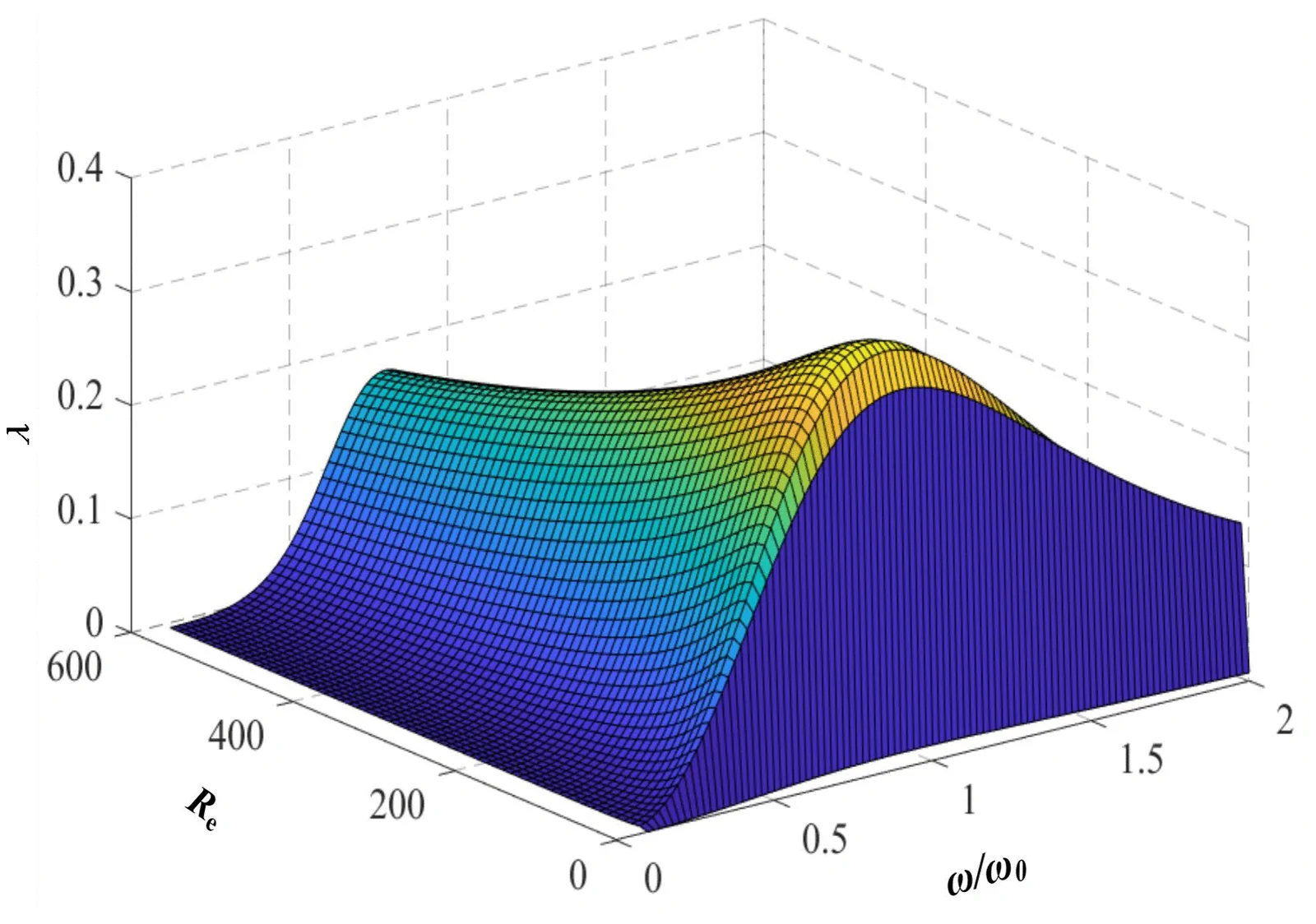
Variation of efficiency-power product with angular frequency and output equivalent impedance.

Through the above analysis, it is found that the output power and transmission efficiency of the S-P type compensation topology IPT system are primarily influenced by the parameters of the transmitter and receiver coil self-inductance, resonant capacitance, coupling coefficient, resonant angular frequency, and equivalent load resistance. This paper primarily aims to optimize the maximum efficiency power product *λ* by selecting appropriate coupling coefficient *k*, resonant angular frequency *ω*, and equivalent load resistance *R*_e_ parameters. This approach enables the IPT system to achieve globally optimal transmission characteristics by comprehensively considering both output power and transmission efficiency metrics.

Under the condition that other parameters remain unchanged, the coupling coefficient is optimized. Table 1 compares the optimization of the coupling coefficient with the objectives of maximizing power and maximizing the efficiency-power product, respectively. In Table 1, it can be seen that when optimizing with the maximum efficiency-power product as the objective, the circuit sacrifices 5.9% of the output power but improves the efficiency by 9.8%. Considering both output power and transfer efficiency, the maximum efficiency-power product method enables the circuit to achieve a globally optimal design.

**Table 1:** Power and efficiency comparison.

Index	*k*	*P*	*η*
Maximum output power	0.421	2892	0.71
Maximum efficiency power product	0.492	2721	0.78
Increase Δ(%)		−5.9	+9.8

To ensure the system achieves the maximum efficiency power product *λ* output target when the coupling coefficient *k* varies, the non-contact transformer can be optimized to alter the coupling coefficient, as referenced in [27,28]. The system achieves the maximum efficiency power product *λ* output target when the inverter angular frequency *ω* varies, which can be realized through frequency modulation control strategies. To maintain the maximum efficiency power product *λ* output target when the equivalent load resistance changes, the actual equivalent load must match the optimal equivalent load *R*_e_opt_. The main circuit of the IPT system is shown in Fig 3. This paper employs a Buck-Boost circuit inserted between the secondary-side rectifier circuit and the load resistor. By adjusting the Buck-Boost circuit, optimal impedance matching is achieved to attain the maximum efficiency-power product *λ* output target.

Based on the operating principle of Buck-Boost converters in continuous current mode (CCM), the relationship between the equivalent impedance Re on the secondary side of the system and the load resistance RL is given by:


$$\begin{array}{c} R_{\text{e}}=\frac{\text{8}}{\pi^{\text{2}}}{\left(\frac{1-D}{D}\right)}^{\text{2}}R_{\text{L}} \end{array}$$ (10)

Where *D* is the duty cycle of the Buck-Boost circuit.

From Eq. (10), it can be seen that by adjusting the duty cycle *D* of the Buck-Boost impedance matching network, the equivalent load resistance Re can be altered. When *R*_e_ = *R*_e_opt_, the system can operate at the maximum efficiency power product λ output target.

## 3. Parameter optimization of IPT systems based on genetic algorithms

This paper combines adaptive genetic algorithms with nonlinear programming to optimize the system, enabling the algorithm to rapidly locate the global optimum solution. Genetic algorithms encode potential solutions to a problem as chromosomes, which undergo multiple generations of evolution (selection, crossover, mutation). The process terminates when defined constraints or iteration limits are met. Simultaneously, it employs a global parallel search to identify the optimal individual within the population, thereby selecting the best candidate as the solution.

The nonlinear programming model for the system first defines the objective function, constraints, and parameters to be optimized. The IPT system adopts the maximum efficiency-power product as its optimization objective function. Considering inverter cost and high-frequency electromagnetic interference factors, the upper and lower limits of the inverter operating angular frequency are determined as *ω*_min_ and *ω*_max_. Considering the distance between primary and secondary coils, offset position, and output power requirements in ICPT system applications, the upper and lower limits of the non-contact transformer coupling coefficient are set as *k*_min_ and *k*_max_. The output power must exceed the system’s rated design power *P*_e_. To minimize system volume, reduce costs, and enhance transmission efficiency, the resonant inductance value should not be excessively large. After defining the constraint ranges for *k* and *ω*, the upper limits *L*_P_h_ and *L*_S_h_ for *L*_P_ and *L*_S_ can be determined based on the actual application requirements for the non-contact transformer’s area and distance, combined with the resonance conditions of the S-P resonant network.

Based on the above analysis, the IPT system adopts the maximum efficiency-power product as its objective function, with the coupling coefficient *k*, resonant angular frequency *ω*, and equivalent load resistance *R*_e_ serving as optimization variables. The nonlinear programming model of the IPT system is described as follows:


$$\begin{array}{c} \left\{ \begin{array}{cc} @ll@max & \lambda \left(\omega ,k,R_{\text{e}}\right)\\ \text{s.t.} & \omega_{min}\leq \omega \leq \omega_{max}\\ & k_{min}\leq k\leq k_{max}\\ & P\left(\omega ,k,R_{\text{e}}\right)\geq P_{\text{e}}\\ & L_{\text{s}}\leq L_{\text{s\_h}}\\ & L_{\text{p}}\leq L_{\text{p\_h}}\\ & C_{\text{1}}=1/\omega_{\text{0}}^{\text{2}}L_{\text{p}}\\ & C_{\text{2}}=1/\omega_{\text{0}}^{\text{2}}L_{\text{s}} \end{array}\right. \end{array}$$ (11)

This paper combines adaptive genetic algorithms with nonlinear programming to optimize the system, enabling the algorithm to rapidly locate the global optimum solution. The system parameter design and optimization process is illustrated in Fig 8. The GA in this paper was compiled and run in the MATLAB environment.

**Fig 8:**
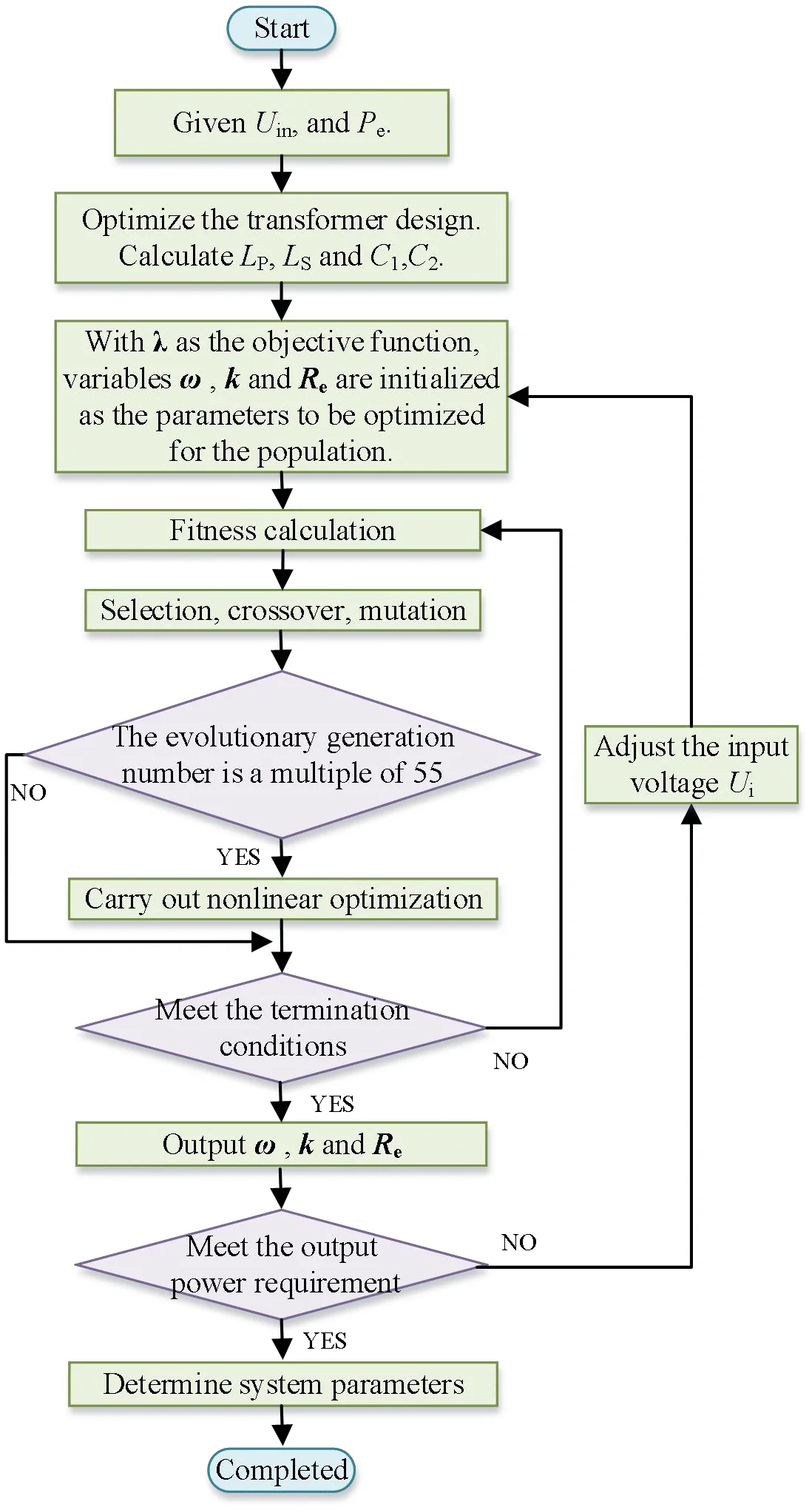
IPT system parameter design and optimization process.

In the genetic algorithm depicted in Fig 8, the coupling coefficient *k*, resonant angular frequency *ω*, and equivalent load resistance *R*_e_ are encoded. In GA, the floating-point encoding method is suitable for representing numbers with a large range, as well as for genetic search in a large space, and also facilitates handling complex decision variable constraints. Therefore, this paper adopts the floating-point encoding method for the optimization variables, and the chromosome of the optimization parameters is described as:


$$\begin{array}{c} X=\left[x_{\text{1}},x_{\text{2}},x_{\text{3}}\right]=\left[k,\omega ,R_{\text{e}}\right] \end{array}$$ (12)

The population size is set to 500, and the number of generations is set to 55. The algorithm uses the condition that the fitness change does not exceed a threshold of 0.02 as the termination criterion, and outputs the current maximum fitness value. The efficiency-power product *λ* serves as the fitness function, where individuals with higher function values exhibit greater fitness and superior quality. Individuals with higher fitness values within the population have a greater probability of being selected. Selected individuals form a new population and continue to reproduce to generate the next generation. The selection operation uses the proportional selection operator, which is a selection method that determines the probability of an individual’s offspring being inherited based on a probability proportional to the fitness of each individual. The probability *P*_i_ that individual i is selected is given by:


$$\begin{array}{c} P_{\text{i}}=\frac{\lambda_{\text{i}}}{\sum_{\text{i}=1}^{\text{n}}\lambda_{\text{i}}}\begin{array}{cc} & \text{i}=1,2,3 \end{array}\ldots \end{array}$$ (13)

Where λ_i_ is the fitness value of individual i.

The crossover process is the main step in generating new individuals throughout the genetic algorithm. Parts of the chromosomes between two individuals are exchanged with a probability of *P*_s._ The value of *P*_s_ cannot be too large, otherwise it will destroy the excellent individuals in the population, but it cannot be too small either, otherwise it will slow down the evolutionary speed of the population. In this paper, the mutation rate during the execution of genetic algorithms is used to maintain population diversity and prevent premature convergence. Therefore, this paper makes a trade-off between premature convergence and slow convergence of the algorithm through the trial-and-error method. When the algorithm is executed, the crossover rate *P*_s_ is selected as 0.7 and the mutation rate as 0.03.

Every 55 generations of genetic algorithm evolution, the obtained local optimal values are used as new individual chromosomes for further evolution. Eq. (11) serves as the fmincon function and its constraints in this optimization algorithm. The termination generation is set to 55, and computation stops when the weighted average change in the fitness function falls below the deviation of the fitness function value. Fig 9(a) shows that the maximum fitness value is reached after 55 iterations.

**Fig 9:**
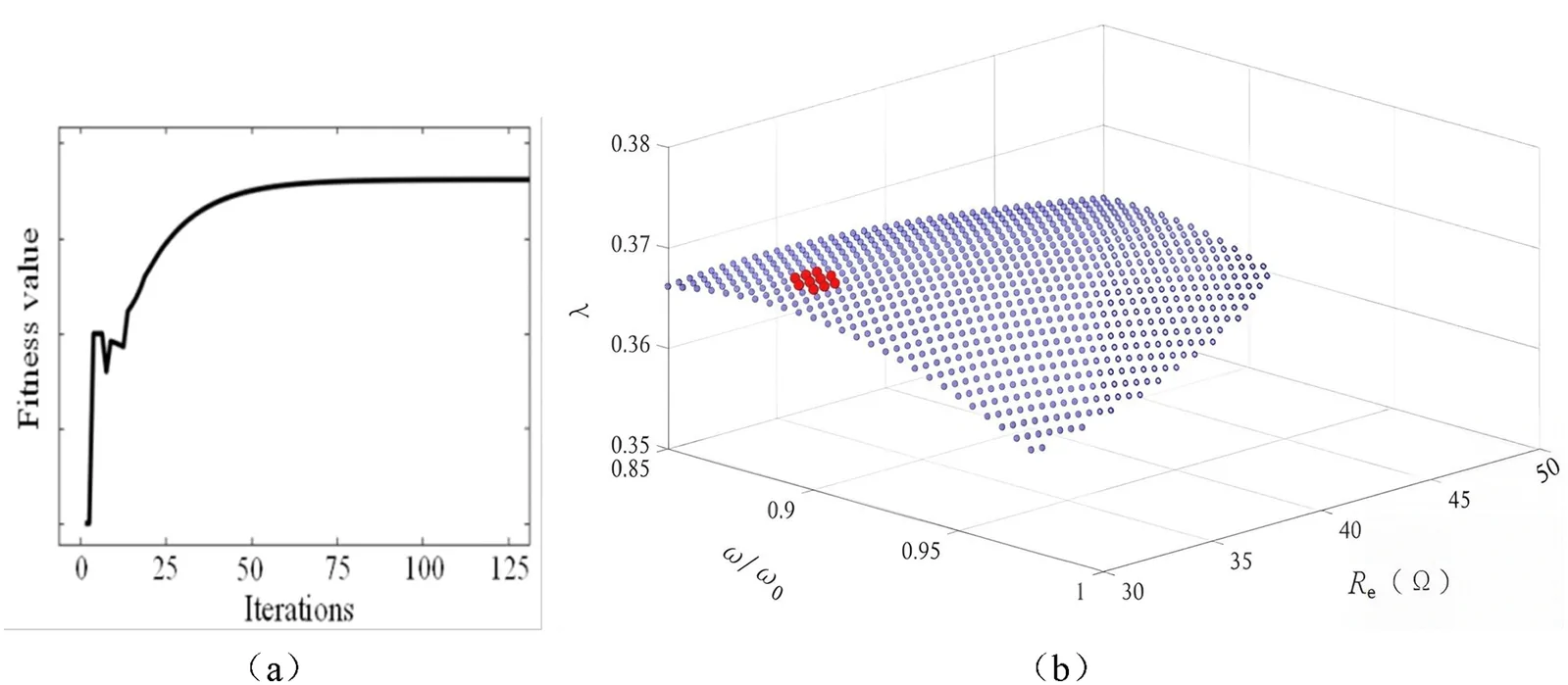
The optimization result of the GA: (a) Convergence curve; (b) Optimal Solution Tracking Plot.

Based on the optimized *k*, *ω*, and *R*_e_ parameters, verify whether the required rated power is met. If not, adjust the input voltage *U*_i_ and restart the optimization algorithm. If the power requirement is satisfied, output the system parameters and terminate the program.

The convergence of the genetic algorithm can be observed in Fig 9(a). Fig 9(b) shows the tracking plot of the optimal parameters *ω*/*ω*₀ and *R*_e_ obtained from a single run of the genetic algorithm with a fixed coupling coefficient k. The densest cluster of red points in the figure represents the optimal solution obtained through the search.

In Table 2, the optimization results for the parameters k, ω, and Re are presented. As shown in the table, the results obtained from each run are not entirely identical, but the performance variations across the system are minimal. The table presents the standard deviations and 95% confidence intervals of the data. Therefore, it is only necessary to select one set of solutions according to the real-time operating state of the system, and then combine it with the resonant network parameter configuration method to determine the other parameters that satisfy the various constraints in the system.

**Table 2:** Optimization parameters of the genetic algorithm.

options	*ω/ω*_*0*_	*k*	*R*_e_	*P*	*η*	λ
**parameters**	0.98	0.49	30	1694	0.9	0.363
	1	0.5	34	1653	0.92	0.362
	1.01	0.48	32	1694	0.9	0.363
	0.97	0.49	29	1675	0.91	0.363
	1.02	0.51	31	1653	0.92	0.362
**Standard deviation**	0.0207	0.0114	1.9235	20.51	0.0100	0.00055
**Confidence intervals(95%)**	[0.9703, 1.0217]	[0.4798, 0.5082]	[28.81, 33.59]	[1648.3, 1699.3]	[0.8976, 0.9224]	[0.3619, 0.3633]

## 4. Studies by the experiments

To validate the theoretical analysis presented in this paper, This research has established a wireless power transmission experimental platform with a maximum output power of 2 kW at full load. It should be noted that this experimental setup is intended solely to verify the correctness and validity of the theoretical analysis. The experimental parameters are listed in Table 3.

**Table 3:** Experimental platform parameters.

Parameter	Number value	Parameter	Number value
*U*_in_/(V)	200	Air gap (mm)	0 ~ 100
*C*_1_/(nF)	141	*L*_s_/(uH)	112
*C*_2_/(nF)	141	*L*_p_/(uH)	112
*k*_min_	0. 326	*k*_max_	0. 52
*f*_min_/(kHz)	35	*f*_max_/(kHz)	45

This study employs a planar U-shaped non-contact transformer. The steady-state magnetic flux density distribution of the planar U-shaped non-contact transformer is depicted in Fig 10. To validate the influence of the coupling coefficient on output power and transmission efficiency, the coupling coefficient was experimentally measured as the lateral and longitudinal distances between the primary and secondary coils varied. The experimental fitting curves of the coupling coefficient versus lateral and longitudinal offset distances are shown in Fig 11. The longitudinal distance between primary and secondary coils varied from 0 to 100 mm, while the lateral distance ranged from 0 to 80 mm. The pre-designed coupling coefficient range is indicated by the pink shaded area in Fig 11, spanning 0.326 to 0.52. The comparative experiments on parameter optimization using genetic algorithms are shown in Fig 12. In the unoptimized system, the output power efficiency exhibits extreme polarization: when the offset distance is very small, the efficiency is high, but the power is low; when the offset distance is very large, the efficiency is low, but the power is high. There is no global consideration of both power and efficiency metrics. After parameter optimization using the genetic algorithm, the system is capable of achieving higher output power and transmission efficiency across a wide range of offset variations. It can also be observed that, to a certain extent, the system optimized by the genetic algorithm achieves a global optimum that balances power and efficiency.

**Fig 10:**
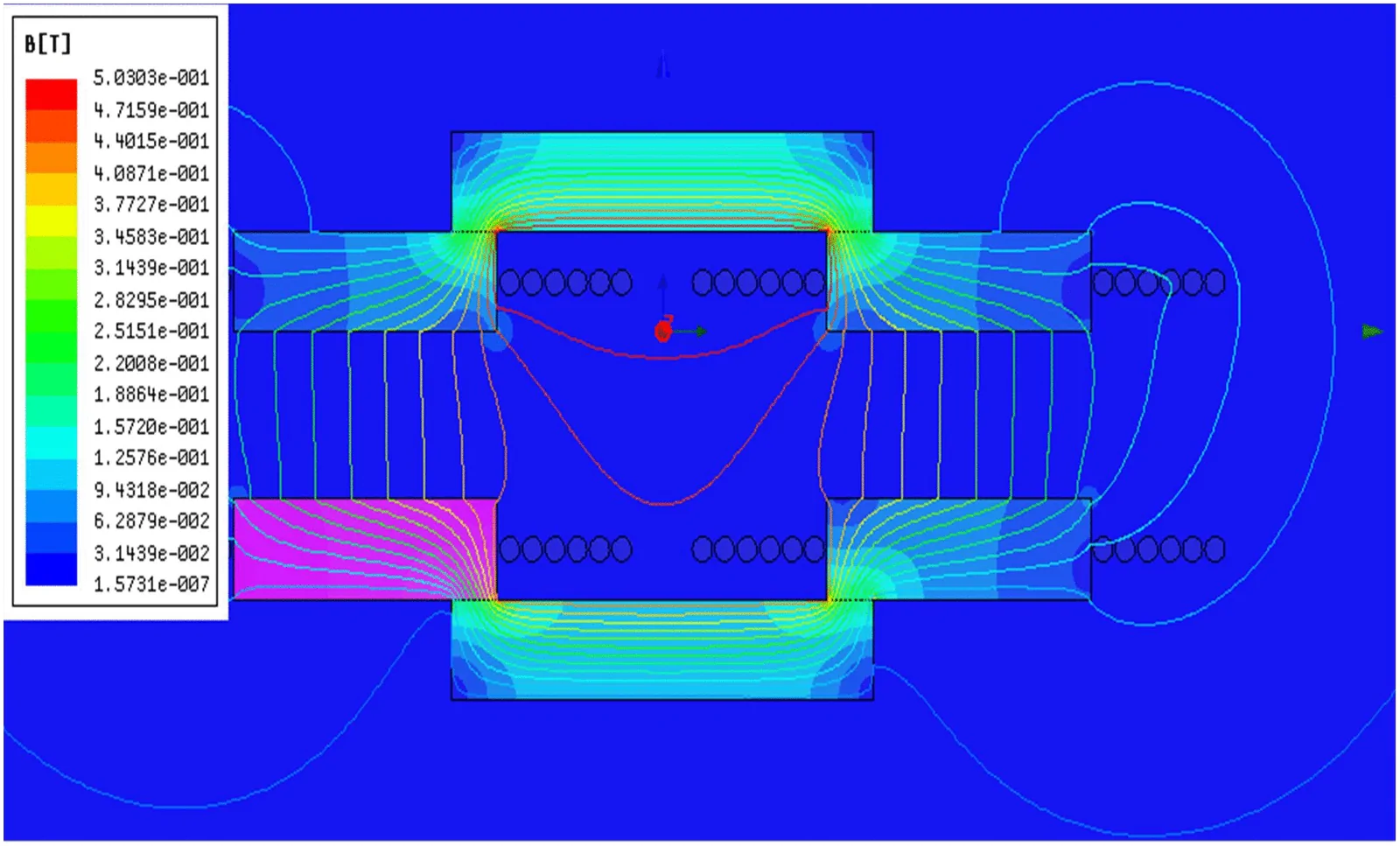
Steady-state magnetic flux density distribution cloud map of a planar U-shaped non-contact transformer.

**Fig 11:**
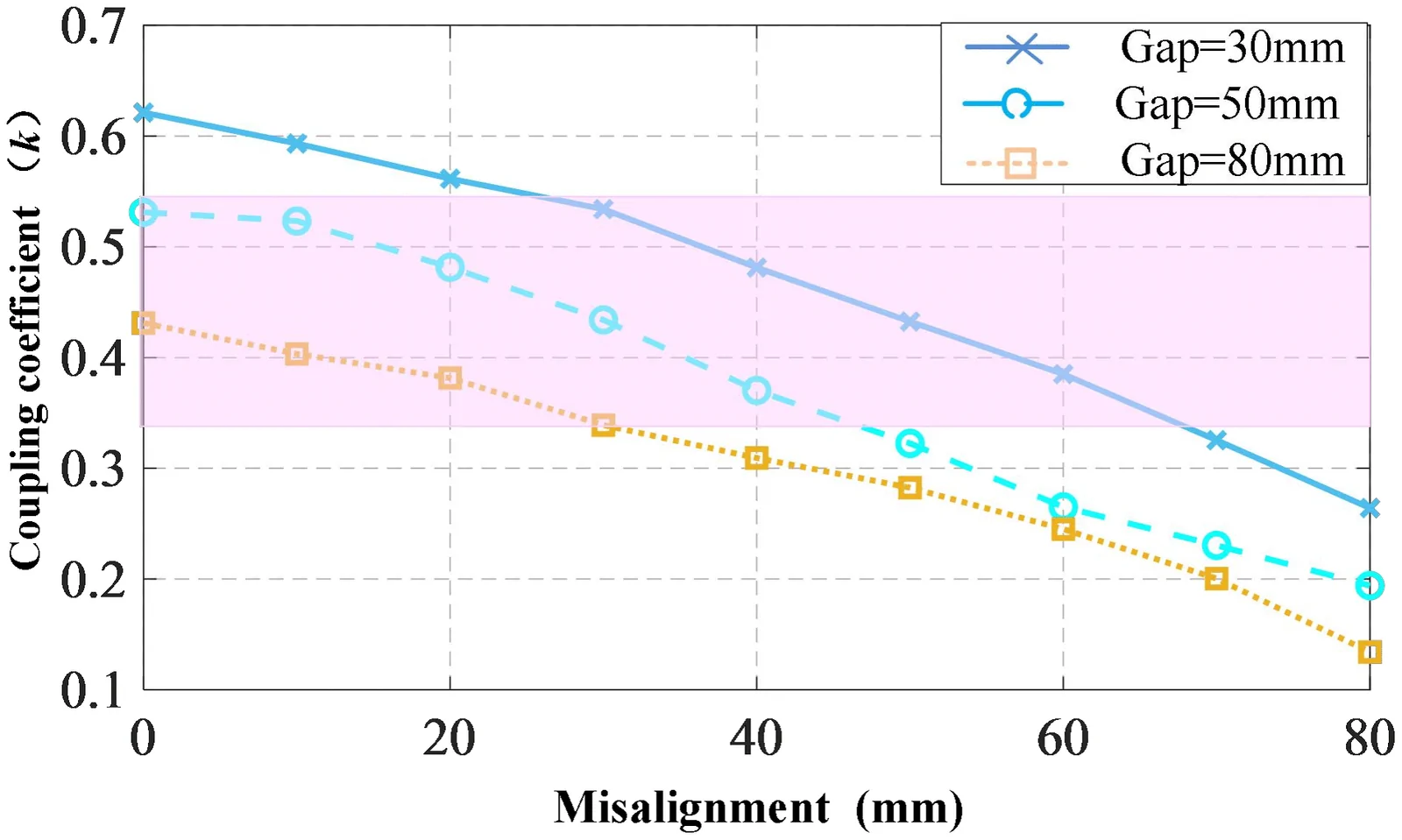
Experimental fitting curve of coupling coefficient vs. lateral displacement distance.

**Fig 12:**
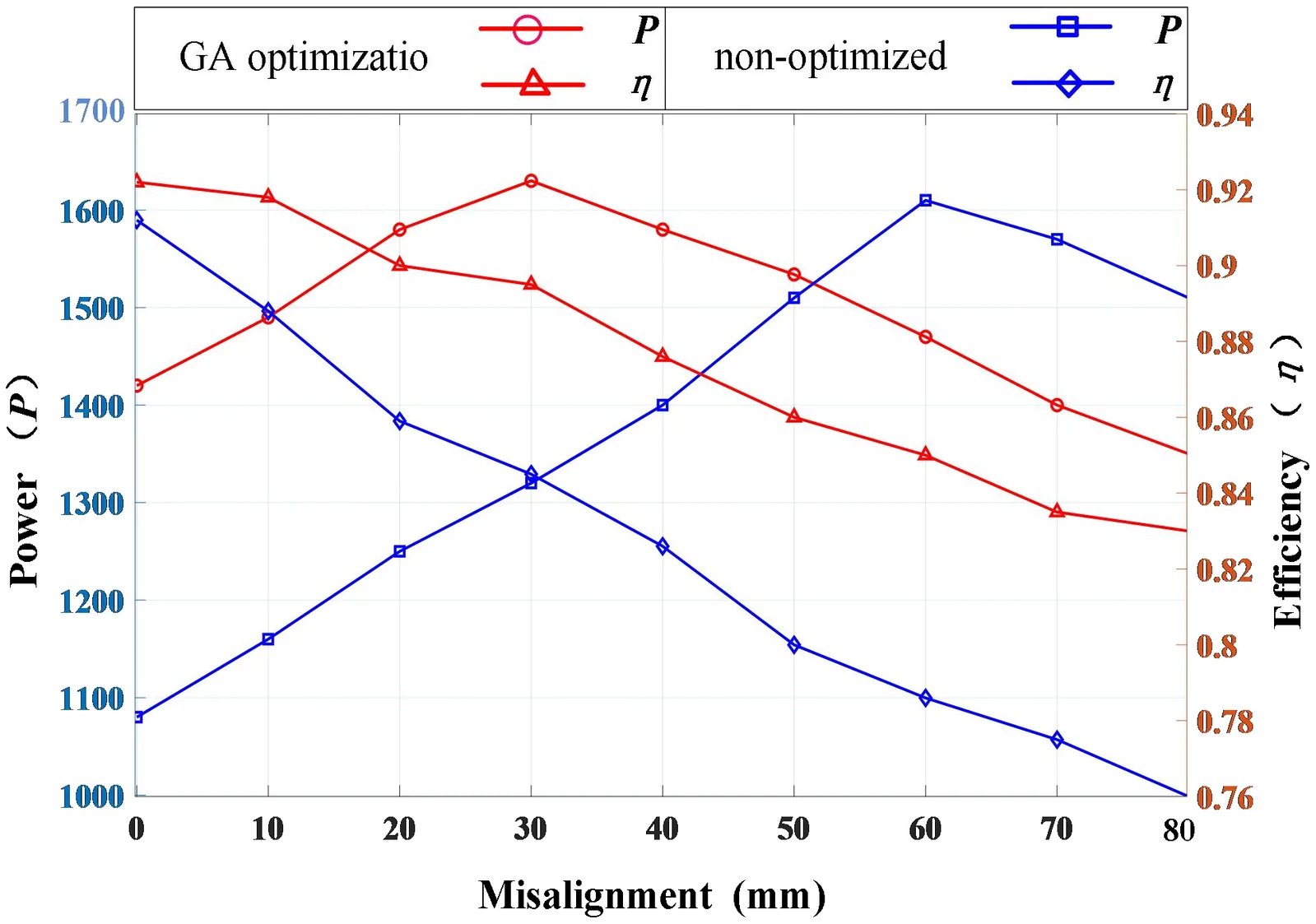
Experimental fitting curve of output power and transmission efficiency as a function of lateral offset distance.

Table 4 compares the main contributions of this work with those of other researchers. In Table 4, it can be seen that other studies are based on impedance matching or coupled transformer model optimization methods, whose main drawback is the strong dependence on system parameters, making them unable to track parameter variations to improve performance metrics. The parameter optimization approaches based on the non-dominated sorting genetic algorithm and multi-objective particle swarm optimization adopt a multi-parameter, multi-objective optimization scheme. Although they consider multiple metrics such as power and efficiency, they increase the complexity. In this paper, by introducing the efficiency-power product as the objective function and performing parameter optimization using a genetic algorithm, a globally optimal design that considers both power and efficiency is achieved.

**Table 4:** Existing performance improvement methods for IPT.

Reference	Improvement objective	proposals	Main contributions or Drawbacks
[11, 12]	Misalignment tolerance improvement	Optimization of a loosely coupled structure model	Dependence on system parameters complex design
[13]	Improve transmission efficiency	Impedance matching	Dependence on system parameters complex design
[21,22]	Improve transmission efficiency	non-dominated sorting genetic algorithm	Only efficiency was considered without taking power indicators into account
[23]	Increase transmission power and efficiency	Multi-objective Particle Swarm Optimization algorithm	Multi-objective parameter optimization influenced by multiple parameters
**This paper**	**efficiency-power product**	**Genetic algorithm**	**Single-objective parameter optimization influenced by multiple parameters** **A global optimal design that takes into account both power and efficiency**

## 5. Conclusion

This paper investigates the S-P type IPT system and establishes an equivalent circuit model for the non-contact transformer mutual inductance. The expressions for the output power and transmission efficiency of the S-P resonant compensation network are derived. The influence characteristics of the coupling coefficient *k*, resonant angular frequency *ω*, and equivalent load resistance *R*_e_ on the circuit output power and transmission efficiency are analyzed. The resonant network parameters of the IPT system are optimized using the efficiency-power product and a genetic algorithm. This approach resolves the challenge of determining parameters for resonant compensation structures. By setting the efficiency-power product as the objective function and *k*, *ω*, and *R*_e_ as decision variables, the paper innovatively transforms the IPT system design problem into a nonlinear parameter optimization problem. This not only enables the fitness function to escape local optima more effectively but also significantly accelerates the algorithm’s convergence speed. The equivalent load resistance is optimized by adjusting the duty cycle of the Buck-Boost circuit. Parameters *k*, *ω*, and *R*_e_ are tuned to achieve the maximum efficiency power product output. Through parameter design, the IPT system attains a globally optimal design for both output power and transmission efficiency.

## Supporting information

S1 Data Minimum dataset. (XLSX)
